# Mid-Term Outcomes of Unstable Complex Proximal Interphalangeal Joint Fracture Management Using the Ligamentotaxor® Device: A Case Series of 33 Cases

**DOI:** 10.7759/cureus.10519

**Published:** 2020-09-17

**Authors:** Amr Abouelela, Islam Mubark, Mohammed Hassan, Michael Howells, Neil Ashwood, Christos Kitsis

**Affiliations:** 1 Trauma and Orthopaedics, University Hospitals of Derby and Burton NHS Foundation Trust, Derby, GBR

**Keywords:** proximal interphalangeal joint, fracture, ligamentotaxis, dynamic, fixator

## Abstract

Background and objective

Fractures of the proximal interphalangeal joint (PIPJ) of the hand have always been difficult to treat, often leading to less than satisfactory outcomes. The use of dynamic external fixator devices to treat these fractures is well established and it is based on the philosophy of minimal soft tissue injury and early joint mobilization. There has been a wide variety in their designs, surgical technique, and reported outcomes. This study aimed to report the long-term outcome following the use of the Ligamentotaxor® device (Ligamentotaxor1, ArexTM, Palaiseau, France) in treating fractures of the PIPJ of the hand.

Methods

Between 2009 and 2018, 33 patients treated in our institution with Ligamentotaxor® for fractures of the PIPJ were followed up for a minimum period of 12 months. Radiographs and clinical records were reviewed for clinical and functional outcomes including finger range of motion (ROM), union, Quick Disability of the Arm, Shoulder, and Hand (QuickDASH) score, and any complications.

Results

A total of 33 patients completed a minimum follow-up of 12 months (mean: 27.5 months). All fractures showed radiological union at a mean of 33 days. Surgery was performed within a mean of 8.9 days and surgical operating time averaged 23.7 minutes. Devices were removed at a mean of 33 days. At the end of the follow-up, the mean range of flexion was 66 degrees and the mean extension lag was six degrees. The mean QuickDASH score was 8.72. Of note, 85% of the patients experienced no limitations in their daily activities, while 35% reported pain on exertion. One patient had a pin tract infection. Four patients had cold intolerance and persistent swelling.

Conclusion

The results of the use of Ligamentotaxor® in this series are comparable to those of other dynamic external fixator devices reported in the literature. Thanks to its quick and easy surgical technique, the device provides an appealing option for the management of PIPJ fractures.

## Introduction

Proximal interphalangeal joint (PIPJ) fractures of the hand are often difficult to manage, often resulting in less than satisfactory outcomes. The notoriety of these fractures is attributed to long-term complications such as stiffness, arthritis, and persistent pain [1]. Anatomic reduction of these fractures has not been shown to correlate with clinical outcomes, and the priority is to achieve well-aligned, reduced joint, and to initiate early range of motion (ROM) to promote cartilage healing [2]. In 1986, Schenck described the first known modern dynamic external fixator for PIPJ fractures [3]. Since then, there has been a vast array of different devices with different surgical techniques, designs, and outcomes. They all share the philosophy of ligamentotaxis where soft tissue tension is used to reduce fractures and distract joint surfaces allowing for early joint mobilization [4,5]. In this study, we report the outcome of using the relatively newly designed Ligamentotaxor® device (Ligamentotaxor1, ArexTM, Palaiseau, France) in treating 33 patients with unstable intraarticular PIPJ fracture-dislocations at a single institution and comparing their outcomes to reports of the outcomes of other dynamic external fixators in the literature.

## Materials and methods

Materials

Between 2009 and 2018, 33 patients with unstable fractures of PIPJ were treated using the Ligamentotaxor® dynamic external fixator at Queens Hospital, Burton. Inclusion criteria were acute injury of less than 10 days' duration and a minimum of 12 months of follow-up. Patients with open fractures and neurovascular and/or tendon injuries were excluded from the study. Fractures with concomitant extended shaft fractures together with articular involvement, which required open reduction and internal fixation in addition to the external fixation device, were also excluded to avoid confounding variables.

**Figure 1:**
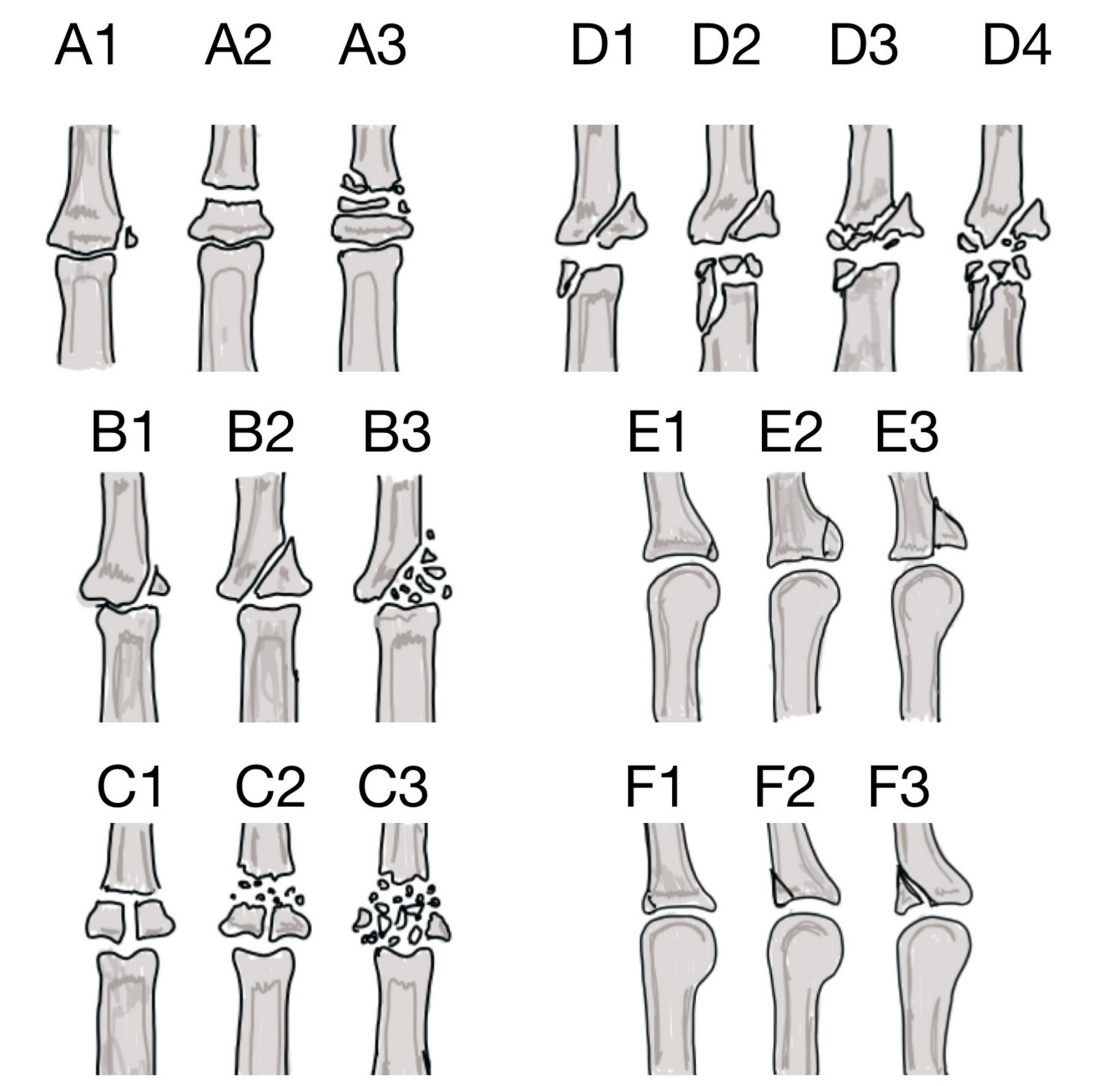
Illustration of different types of proximal interphalangeal joint fractures as per Pélissier’s classification A: extra-articular fractures. B: simple articular fractures. C: pilon fractures. D: fracture involving both ends of the joint. E: palmar lip fractures. F: dorsal lip fractures The fractures are further graded from 1 (one fragment) to 3 (comminuted)

A total of 33 patients were eligible for inclusion in the study. Fractures were classified according to Pélissier’s classification based on the initial radiographs (Figure 1) [6].

In this classification, joint fractures are labeled as A (extra-articular fractures), B (simple articular fractures), C (pilon fractures), or D (fracture involving both ends of the joint) and are graded from 1 (one fragment) to 3 (comminuted). Two other categories, E (corresponding to palmar lip fractures) and F (corresponding to dorsal lip fractures), were added later [7]. E type and B type fractures represented most of the cases with 33% each, and type C was the third most common with 21%. All patients were operated on by one of the two senior authors, CK and NA, under general anaesthesia with or without a regional block. All cases were performed as a day case surgery.

Operative technique

**Figure 2:**
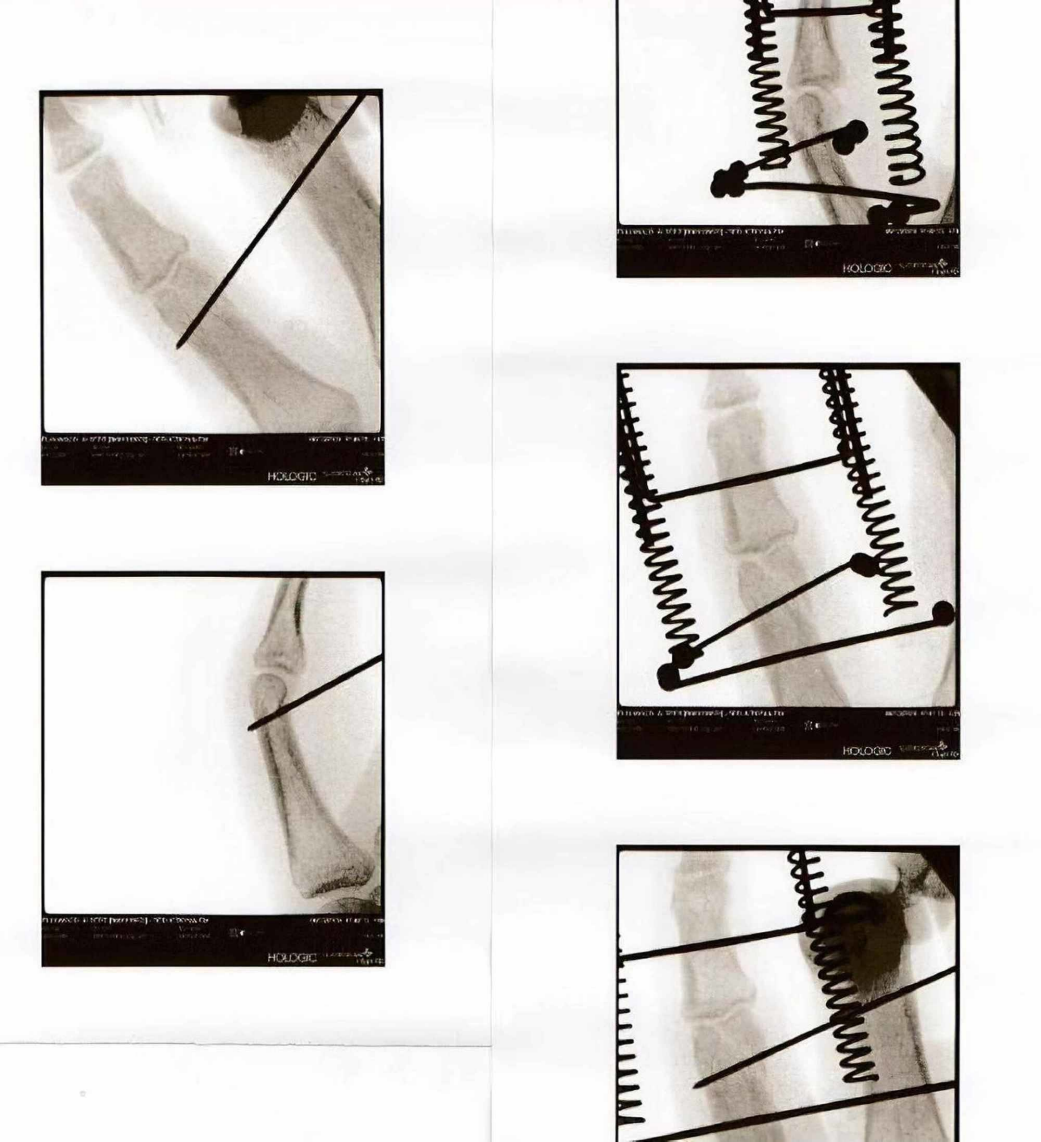
Intraoperative fluoroscopy imaging demonstrating Ligamentotaxor® device installment and fracture reduction

The Ligamentotaxor® pack includes two 1.2-mm wires, two plastic rods, two 0.8-mm diameter springs, and a third 1.5-mm diameter U-shaped pin. The proximal 1.2-mm wire was placed perpendicular to the finger axis approximately at the center of the condyles of the proximal phalanx in cases of middle phalanx base fractures, or proximally in the shaft of proximal phalanx as close as possible to the base of the phalanx, without compromising the adjacent web in cases of head proximal phalanx fractures [8-12]. The two plastic rods were then fitted to the proximal wire. The proximal wire was then bent to 90 degrees, cut short, and capped. The distal 1.2-mm wire was placed in the middle phalanx parallel to the proximal wire using an external guide and was kept as far as possible from the proximal pin in cases of base middle phalanx fractures, or as close as possible to the axis of the joint in cases of head proximal phalanx fractures [8-12]. Again, this wire was bent to 90 degrees in the same coronal plane as the phalanx. The two springs were then railed over the bent wire and plastic rod on each side. The springs were screwed towards the proximal wire until satisfactory fracture reduction and joint distraction were achieved, approximately 1-2 mm, under fluoroscopy control (Figure 2). The 1.5-mm U-shaped pin was then railed in the two springs to add stability to the frame and counter axial rotation.

Rehabilitation protocol

**Figure 3:**
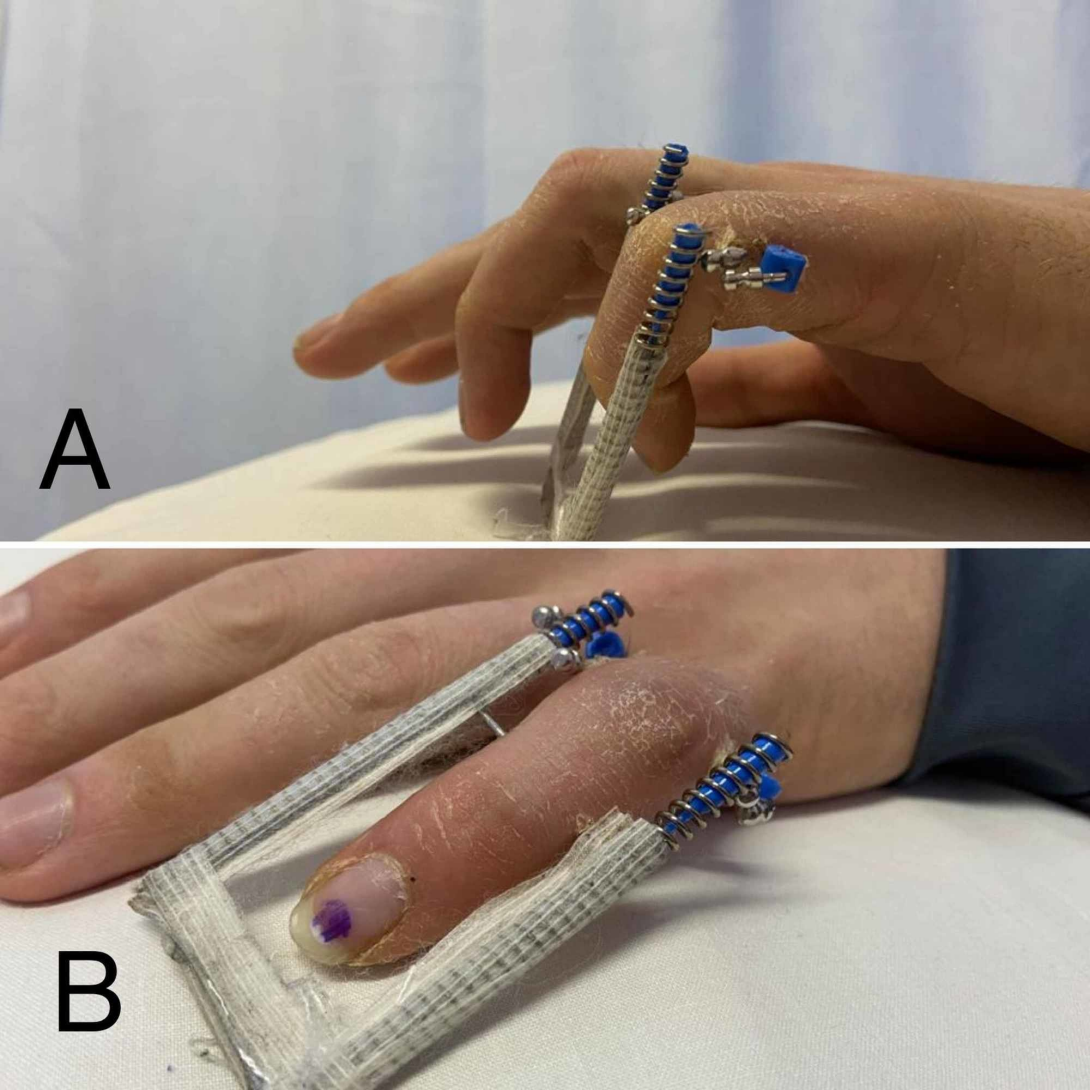
Clinical photographs demonstrating an active range of motion with the device installed during the follow-up visits

Patients were seen the following day in the hand therapy clinic for the start of the mobilization procedure. The protocol included an initial two weeks of passive ROM followed by active ROM until the removal of the frame (Figures 3A, 3B).

Plain radiographs were obtained at two- and four-weeks’ time to check for fracture healing and joint alignment. Frames were usually left for four to five weeks and all frames were removed in the outpatient clinic. Follow-up assessments in terms of recording PIPJ ROM were performed by the physiotherapist during the follow-up appointments before discharge and the Quick Disability of the Arm, Shoulder, and Hand (QuickDASH) scores were collected by post in the form of filled-in reports by patients or over the phone. Fracture healing and joint alignment were evaluated on radiographic examinations. Any complications related to surgery or fracture were also recorded.

Case example

**Figure 4:**
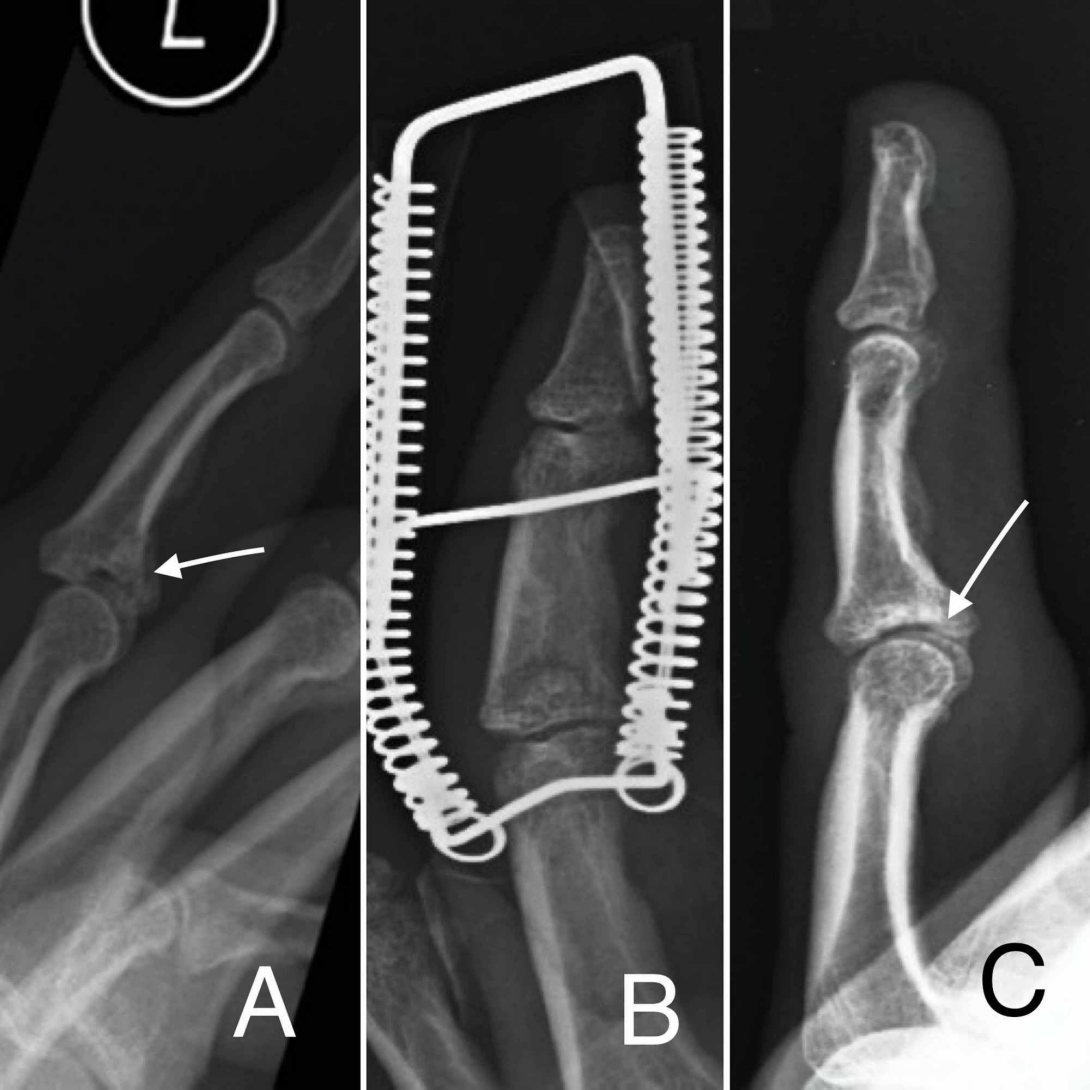
Radiographic images at various stages A: preoperative radiograph demonstrating E2 type fracture of the base of the middle phalanx left ring finger. B: two-week postoperative radiograph demonstrating fracture reduction using the Ligamentotaxor® device. C: six-month postoperative radiograph demonstrating fracture healing and restoration of joint congruency

A 36-year-old female presented with E2 fracture of the base of the middle phalanx left ring finger; six days later, she had a fixation with Ligamentotaxor® and removed 26 days later; ROM at the end of follow-up was 0-70 degrees and the QuickDash score was 0. The patient reported cold intolerance (Figures 4A-4C).

## Results

A total of 33 patients who satisfied the inclusion criteria were included in the result analysis: 23 men and 10 women with a mean age of 36 years (range: 18-70 years). The mechanism of injury was a contact-sports injury in 70% of the cases while the remaining 30% was sustained during work. The mean time to surgery was 8.9 days (range 1-10 days), and the average operating time was 23.7 minutes. Ligamentotaxor® was removed at a mean of 33 days postoperatively. The mean of the follow-up duration was 27.5 months (range: 12 to 108 months) with a median of 34 months. At the end of the follow-up, the mean QuickDASH score was 8.716 (range: 0 to 27.63); 85% of the patients had no limitations in their daily activities and 35% experienced pain on exertion. The mean PIPJ flexion was 66 (range: 15 to 90), and the extension lag averaged 6 (range: 0 to 20). Union was achieved in all cases at a mean of 33 days postoperatively.

The results, in terms of QuickDASH score and ROM, varied from good in less serious fractures to worse in complex ones. Less favorable results were reported in type C fractures; unfortunately, given the small number of cases and the vast diversity of fracture types, statistical analysis was not performed. There was no statistically significant difference between fractures of the head proximal phalanx and those of the base of the middle phalanx, which can be attributed to a small number (four cases) of head proximal phalanx fractures. One patient developed a superficial pin tract infection that responded well to oral antibiotics. Cold intolerance and persistent swelling were reported in four cases. Two patients developed radiological signs of arthritis at 36 and 40 months respectively, which can be attributed to suboptimal treatment related to residual central depression of the articular surface in one of the patients and failure to attend early physiotherapy appointments in the other patient while both patients were in their forties, even though they had optimal pin placement, timing of surgery, and pin removal. Neither of the two patients required further surgery.

## Discussion

Fracture dislocations of PIPJ can result in a great loss of ROM with a subsequent disability of hand function. Suboptimal treatment can result in long-term complications such as pain, arthritis, and deformity [13,14]. There is a vast array of treatment options for this injury, including closed reduction, open reduction, percutaneous wire fixation, external fixation, extension block techniques, and hemi-hamate arthroplasty [15]. In a recent systematic review by Demino et al., no particular treatment method or fracture type yielded consistently better outcomes over another [16]. Essentially, any treatment option should aim to reduce edema, prevent tendinous adhesions and joint stiffness, and facilitate rehabilitation through early active mobilization. Moreover, some authors have shown that anatomic restoration of joint surfaces is not needed to achieve satisfactory results [17].

Since Schenck’s work in 1986, there has been renewed interest in the use of dynamic external fixation of these injuries [3]. Dynamic external fixator devices utilize the concept of ligamentotaxis to reduce fracture and dislocation, allow for the early mobilization of the joint while avoiding risks associated with open surgery, such as edema, scar tissue formation, and stiffness [4,5]. These devices have different designs and methods of application, ranging from simple pins and rubber band system to more bulky complex compass hinge devices. However, reported outcomes have not shown that a certain device is superior to another [18,19]. Therefore, it is rational to favor devices that are more cost-effective, easy to apply, and less cumbersome to the patient.

The Ligamentotaxor® system is relatively straight forward to construct in a timely manner. The flexibility of the spring coil allows for movement at the PIPJ even with the proximal wire not perfectly in the center of the proximal phalanx condyle. It also allows for adjustable distraction even postoperatively regardless of the position of the wires.

In a multicentre study on using Ligamentotaxor®, Pélissier et al. reported a mean ROM of 81° (range: 0-115°) in 88 patients [7]. In another study, Damret et al. reported a mean flexion mobility of 73° (range: 60-100°), and the extension deficit was 13° (range: 0-20°), while Kostoris et al. reported less ROM at 60.5°, mean extension lag of 18.5°, and mean QuickDASH score of 21.6 [20,21]. These results are comparable to those reported on other dynamic external fixation devices. In their systemic review, Demino et al. reported a mean ROM of 81.7 for all dynamic external fixation studies included in their review [16].

The result of the present work shows favorable outcomes with a mean flexion of 66° and a QuickDASH score of 8.72, which is similar to those reported by Pélissier et al. [7], which is so far the best result reported in the literature regarding the use of this particular device. It is our view that Ligamentotaxor® achieves good clinical and functional outcomes by using a relatively easy technique; moreover, it is a low-profile device with a low incidence of complications.

This study has some inherent limitations related to its retrospective design. The was no control group, and the presence of such a group managed with using open reduction internal fixation would have improved the quality and validity of the current study. Further studies involving a larger number of patients would be required to validate these outcomes. The short duration of the minimum follow-up period (12 months) did not allow us to address long-term complications such as post-traumatic arthritis in some patients.

## Conclusions

The results of this study were favorable and consistent with the good outcomes reported in similar studies in the literature. Based on our findings, the Ligamentotaxor® device is simple and quick to apply, and it allows for adjustable traction and early active ROM of PIPJ.
